# Enterohemorrhagic *E. coli* (EHEC) pathogenesis

**DOI:** 10.3389/fcimb.2012.00090

**Published:** 2012-07-12

**Authors:** Y Nguyen, Vanessa Sperandio

**Affiliations:** ^1^Department of Microbiology, The University of Texas Southwestern Medical CenterDallas, TX, USA; ^2^Department of Biochemistry, The University of Texas Southwestern Medical CenterDallas, TX, USA

**Keywords:** EHEC, LEE, acid resistance, cattle, colonization

## Abstract

Enterohemorrhagic *Escherichia coli* (EHEC) serotype O157:H7 is a human pathogen responsible for outbreaks of bloody diarrhea and hemolytic uremic syndrome (HUS) worldwide. Conventional antimicrobials trigger an SOS response in EHEC that promotes the release of the potent Shiga toxin that is responsible for much of the morbidity and mortality associated with EHEC infection. Cattle are a natural reservoir of EHEC, and approximately 75% of EHEC outbreaks are linked to the consumption of contaminated bovine-derived products. This review will discuss how EHEC causes disease in humans but is asymptomatic in adult ruminants. It will also analyze factors utilized by EHEC as it travels through the bovine gastrointestinal (GI) tract that allow for its survival through the acidic environment of the distal stomachs, and for its ultimate colonization in the recto-anal junction (RAJ). Understanding the factors crucial for EHEC survival and colonization in cattle will aid in the development of alternative strategies to prevent EHEC shedding into the environment and consequent human infection.

## Introduction

Verocytoxin-producing *Escherichia coli* (VTEC), also known as Shiga-toxin producing *E. coli* (STEC), is a food-borne zoonotic agent associated with outbreaks worldwide that poses a serious public health concern. Over 380 different VTEC serotypes have been isolated from humans and animals, but only a small number of serotypes are linked to human disease. Serotype O157:H7 is the major source of *E. coli* food poisoning outbreaks in the United States (US) (Karmali et al., 2010). Characteristics of *E. coli* serotype O157:H7 (EHEC) infection includes abdominal cramps and bloody diarrhea, as well as the life-threatening complication hemolytic uremic syndrome (HUS) (Karmali et al., 1983; Karmali, 1989; Griffin and Tauxe, 1991). Karmali and colleagues first identified VTEC as the infectious agent responsible for HUS after correlating *E. coli* infection in patients with diarrhea and HUS with the presence of a toxin that produced significant irreversible cytotoxic effects in Vero cells (Konowalchuk et al., 1977; Karmali et al., 1985). O'Brien and LaVeck later purified the toxin from an enteropathogenic strain of *E. coli* and determined that the toxin was structurally and antigenically similar to the Shiga toxin produced by *Shigella dysenteriae* type 1 (O'Brien and LaVeck, 1983).

Shiga toxin is composed of two major subunits, designated A and B (O'Brien et al., 1992; Paton and Paton, 1998). The B subunit forms a pentamer that binds to globotriaosylceramide-3 (Gb3) (Lingwood et al., 1987), and this specificity determines where Shiga toxin mediates its pathophysiology. The A subunit exhibits an RNA N-glycosidase activity against the 28S rRNA (Endo et al., 1988) that inhibits host protein synthesis and induces apotosis (Sandvig, 2001; Karmali et al., 2010). In humans, EHEC colonizes the large intestine (Phillips et al., 2000). Shiga toxin released by EHEC binds to endothelial cells expressing Gb3, allowing absorption into the bloodstream and dissemination of the toxin to other organs (Sandvig, 2001). The tissues and cell types expressing Gb3 varies among hosts, and the distribution of Gb3 targets the pathology of toxin-mediated disease to cells expressing Gb3 (Pruimboom-Brees et al., 2000). For example, renal glomerular endothelium expresses high levels of Gb3 in humans, and Shiga toxin production results in acute renal failure, thrombocytopenia, and microangiopathic hemolytic anemia, all typical characteristic of HUS (Karmali et al., 1983).

Currently no treatment is available for EHEC infections (Goldwater and Bettelheim, 2012). The use of conventional antibiotics exacerbates Shiga toxin-mediated cytotoxicity. In an epidemiology study conducted by the Centers for Disease Control and Prevention, patients treated with antibiotics for EHEC enteritis had a higher risk of developing HUS (Slutsker et al., 1998). Additional studies support the contraindication of antibiotics in EHEC infection; children on antibiotic therapy for hemorrhagic colitis associated with EHEC had an increased chance of developing HUS (Wong et al., 2000; Zimmerhackl, 2000; Safdar et al., 2002; Tarr et al., 2005). Antibiotics promote Shiga toxin production by enhancing the replication and expression of *stx* genes that are encoded within a chromosomally integrated lambdoid prophage genome. *Stx* induction also promotes phage-mediated lysis of the EHEC cell envelope, allowing for the release and dissemination of Shiga toxin into the environment (Karch et al., 1999; Matsushiro et al., 1999; Wagner et al., 2002).

Cattle are a major reservoir of EHEC, but unlike in humans, EHEC colonization in adult ruminants is asymptomatic (Cray and Moon, 1995; Brown et al., 1997; Dean-Nystrom et al., 1997; Woodward et al., 1999; Wray et al., 2000). While humans express Gb3 on their vascular endothelium that promotes much of the pathophysiology associated with Shiga toxin, cattle lack vascular expression of Gb3 (Pruimboom-Brees et al., 2000). Although Gb3 receptors are detected in the kidney and brain of cattle, Shiga toxin was unable to bind to the blood vessels in the cattle gastrointestinal (GI) tract (Pruimboom-Brees et al., 2000). As a result, Shiga toxin cannot be endocytosed and transported to other organs to induce vascular damage in cattle. In contrast to humans where EHEC colonizes in the colon and causes electrolyte imbalances, EHEC colonizes the recto-anal junction (RAJ) of cattle where it is impervious to the effects of Shiga toxin (Naylor et al., 2003). The insensitivity to Shiga toxin and differential preference in colonization sites make cattle a more tolerant host for EHEC and may contribute to persistence and transmission of this human pathogen.

Cattle transmit EHEC to humans by shedding the pathogen in their feces. Fecal shedding may be brief or more extended (Rice et al., 2003). A proportion of positive animals called “super shedders” excrete more EHEC than others. Although the “super shedders” comprise a small ratio of cattle, it has been estimated that they may be responsible for over 95% of all EHEC bacteria shed (Omisakin et al., 2003; Chase-Topping et al., 2007). Evidence supports that high concentrations of EHEC in feces or prolonged shedding may result from intimate colonization at the RAJ (Cobbold et al., 2007). Once shed into the environment, humans acquire EHEC by consuming contaminated bovine-derived products such as meat, milk, and dairy products (Armstrong et al., 1996) or contaminated water, unpasteurized apple drinks, and vegetables (Cody et al., 1999; Hilborn et al., 1999; Olsen et al., 2002). Direct contact with ruminants at petting zoos or through interactions with infected people within families, daycare centers, and healthcare institutes represent another source of EHEC transmission (Spika et al., 1986; Carter et al., 1987; Rowe et al., 1993; Rangel et al., 2005). Bovine manure can harbor viable EHEC for more than seven weeks (Wang et al., 1996), and the long-term environmental persistence of EHEC poses an increased risk for transmission of EHEC through the fecal-oral route through wash-off to nearby farms or in contaminated grass consumed by other cattle. By gaining a better understanding of how EHEC colonizes the cow, methods can be devised to limit fecal shedding of EHEC into the environment and limit sources of contamination and consequent human infection.

## Factors important for EHEC survival and colonization in cattle

### Acid resistance systems

EHEC adapts an oral-fecal lifestyle in cattle and other ruminants. After being ingested, EHEC enters the rumen of cattle. In order to reach the RAJ for colonization, EHEC must first breach the acidic barrier of the stomachs. EHEC has an intricate acid resistance (AR) system that enables it to survive through the acidic environment of the stomach, as exemplified by its low infectious dose of 10–100 colony-forming units (Tuttle et al., 1999). Three important AR systems have been identified in *E. coli*: the AR system 1 (glucose-repressed or oxidative), AR system 2 (glutamate-dependent), and AR system 3 (arginine-dependent). The relative importance of each AR system *in vivo* is still being delineated; however, induction and function of these systems *in vitro* varies depending on the type of culture medium used and growth conditions (Lin et al., 1995, 1996; Hersh et al., 1996).

Among the three AR systems, the mechanism of glucose-repressed AR system is the least understood. The glucose-repressed AR system is activated in stationary phase in Luria-Bertani broth (LB) and repressed by addition of glucose to culture media. Activation of the glucose-repressed AR system depends on two global regulators: the cAMP receptor protein (CRP), and the stress response alternative sigma factor RpoS. (Castanie-Cornet et al., 1999). Calves inoculated with equal numbers of wild type EHEC and an *rpoS* mutant strain shed the *rpoS* mutant significantly less than the wild type, indicating an important role for RpoS and the glucose-repressed AR system in passage through the GI tract of cattle (Price et al., 2000). Since RpoS is a global stress regulator, eliminating this transcription factor may have other pleiotropic effects that can alter the ability of EHEC to colonize the host.

**Figure 1:**
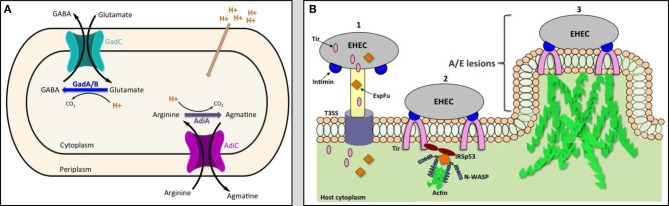
**The model of acid resistance system 2 and 3 (A) and the schematic diagram of the formation of attaching and effacing (A/E) lesions (B).** EHEC injects effector proteins such as Tir and EspFu into the host cytoplasm through the T3SS (1). Tir localizes to the host membrane and binds to intimin to intimately attach the bacteria to the cell. Tir and EspFu recruit host factors (2) to subvert host cytoskeleton and actin polymerization (3).

The glutamate-and arginine-dependent AR systems have similar modes of action (Figure 1). The glutamate decarboxylases GadA and GadB and the arginine decarboxylase AdiA convert glutamate or arginine to γ-amino butyric acid (GABA) or agmatine, respectively, by displacing the α-carboxyl group of the amino acids with a proton that is transported from the environment into the cytoplasm. GABA and agmatine are exchanged for new amino acids through their cognate antiporters GadC and AdiC, respectively (Hersh et al., 1996; Castanie-Cornet et al., 1999). The uptake of the protons increases the internal pH and helps maintain pH homeostasis.

Regulation of the glutamate-dependent AR system is complex and varies with different environmental conditions (detailed review in Foster, 2004). Of the three AR systems, the glutamate-dependent AR system provides the highest level of acid protection (Lin et al., 1996; Castanie-Cornet et al., 1999). Additionally, Price et al. demonstrated that among the three AR systems, glutamate-dependent AR system is necessary for passage through the acidic stomachs and colonization in cattle. Interestingly, the glutamate-dependent AR system was not required for EHEC survival in acidic foods such as apple cider. Instead EHEC utilizes the glucose-repressed AR system to withstand the acid challenge when stored in foods containing low pH (Price et al., 2004). This versatility allows EHEC to utilize different AR systems to persist in diverse environments. Further investigation into the mechanisms EHEC uses to activate the AR systems in cattle will be useful for developing new techniques to reduce EHEC survival through the acidic stomachs and its subsequent colonization at the RAJ.

## Formation of attaching and effacing lesions on epithelial cells

After passage through the acidic barrier, EHEC forms attaching and effacing (A/E) lesions on the mucosal epithelium at the RAJ, allowing for its colonization at the RAJ. A/E lesions are characterized by destruction of microvilli, intimate attachment of the bacteria to the cell, and accumulation of polymerized actin beneath the site of bacterial attachment to form a pedestal-like structure cupping individual bacteria (Figure 1) (Nataro and Kaper, 1998). The genes required for formation of A/E lesions are encoded within the chromosomal pathogenicity island known as the locus for enterocyte effacement (LEE) (McDaniel et al., 1995; Elliott et al., 1998). The LEE consists of approximately 41 genes, divided into five major operons (*LEE1-5*), that encode for a type 3 secretion system (T3SS), regulators, chaperones, and effector proteins. The LEE-encoded regulator (Ler), the first gene encoded in *LEE1*, acts as the master transcription factor of the pathogenicity island, regulating expression of the entire LEE (Elliott et al., 1998; Muller et al., 2009).

The structure of the T3SS resembles a “molecular syringe” where EHEC can inject effector proteins through the T3SS needle directly into the cytoplasm of the target cells. One important secreted protein that is injected into the host is the translocated intimin receptor (Tir). Once released into the host cytoplasm, Tir is directed to the host cytoplasmic membrane and is inserted as a hairpin-like structure, with its N- and C-terminus in the cytoplasm and central domain exposed to the surface. The central domain of Tir interacts with the LEE-encoded surface protein intimin to form a tight attachment of the bacteria to the eukaryotic cell (Kenny et al., 1997; Deibel et al., 1998). Another non-LEE encoded effector protein, *E. coli* secreted protein F-like protein from prophage U (EspFu), is secreted into the cell and works co-operatively with Tir to recruit host proteins to subvert host cytoskeleton and actin polymerization. EspFu recruits actin nucleation-promoting factor Wiskott-Aldrich syndrome protein (N-WASP) and insulin receptor tyrosine kinase substrate p53 (IRSp53), an important regulator for actin cytoskeleton reorganization. This results in accumulation of actin beneath attached bacteria, forming the characteristic pedestal-like structure (Figure 1) (Campellone et al., 2004; Weiss et al., 2009).

*In vitro* studies demonstrate the crucial role A/E lesion formation plays in EHEC attachment to cultured cells. Various groups have investigated whether the formation of A/E lesions is also required for EHEC to attach to bovine intestinal epithelial cells to promote colonization in cattle. Immunofluorescence staining of tissues reveals that EHEC tightly adheres predominately to the epithelial cells in the RAJ of cattle (Naylor et al., 2003). Dziva et al. used signature-tagged transposon mutagenesis (STM) to identify EHEC genes required for colonization and survival in cattle. Transposon insertions in the genes encoding for the T3SS machinery resulted in reduced fecal shedding of EHEC (Dziva et al., 2004). Similarly, deletion of the *LEE4* operon, which encodes for essential structural components of the T3SS, resulted in reduced EHEC ability to colonize cattle (Naylor et al., 2005). These data suggest that the secretion apparatus is important for colonization in cattle. Tir and intimin have also been shown to play an important role in intestinal colonization in neonatal calves and piglets (Donnenberg et al., 1993; McKee et al., 1995; Tzipori et al., 1995; Dean-Nystrom et al., 1998) and in adult cattle and sheep (Cornick et al., 2002). Together the data indicate that LEE-mediated adherence of EHEC to intestinal epithelia is important for promoting colonization in cattle.

In recent years, several non-LEE encoded effectors—EspJ, NleB, NleE, NleF, and NleH—also have been shown to influence EHEC survival and colonization. Although EspJ is not required for A/E lesion formation in HEp-2 cells or human intestinal explants, *in vivo* studies in mice show that EspJ aids in the passage of EHEC through the host's intestinal tract, suggesting a role for EspJ in host survival and pathogen transmission (Dahan et al., 2005). The mouse pathogen *Citrobacter rodentium*, which shares homology of many virulence factors with EHEC, had reduced colonization of *nleB*, *nleH, nleF* mutants in mice compared to the wild-type strain (Kelly et al., 2006; Echtenkamp et al., 2008; Garcia-Angulo et al., 2008). Wild-type EHEC also outcompeted the *nleF* mutant in gnotobiotic piglets for colonization of the piglet colon and RAJ (Echtenkamp et al., 2008). Co-infection of lambs with wild-type EHEC and an *nleH* mutant demonstrated a competitive advantage of the wild-type strain over the mutant (Hemrajani et al., 2008). In contrast, Hemrajani et al. found that the *nleH* mutant colonized the bovine gut more efficiently than wild-type EHEC. While studies in mice and other animal models provide insight into the roles of EHEC virulence genes, further studies are required to evaluate the role that these EHEC effectors perform in cattle.

## Regulation of acid fitness and LEE genes by quorum-sensing

Acid resistance and formation of A/E lesions are crucial for EHEC to establish a persistent oral-fecal lifestyle in cattle. Elucidating the mechanisms by which EHEC regulates these two systems in its natural reservoir provides insight for developing better preventative strategies to reduce EHEC carriage. Progress toward understanding how EHEC regulates both acid resistance and the LEE genes was made recently with the discovery that the transcriptional regulator SdiA regulates both transcription of the LEE genes for A/E lesion formation (Kanamaru et al., 2000; Hughes et al., 2010) and the *gad* genes for acid resistance in cattle (Kanamaru et al., 2000; Hughes et al., 2010). A member of the LuxR family of transcription factors, SdiA senses acyl-homoserine lactones (AHLs) produced by other bacteria.

Bacteria coordinate their behavior through the production and sensing of chemical signals, a mechanism termed quorum-sensing (Fuqua et al., 2001). The LuxR/I system in *Vibrio fischeri* represents the prototypical quorum-sensing system (Nealson et al., 1970; Nealson and Hastings, 1979). Briefly, through LuxI, *V. fischeri* synthesizes AHLs, which diffuse freely across the bacterial membrane into the environment. When the bacterial population reaches a sufficient density, AHLs diffuse back into the bacterial cytoplasm where they bind to the transcriptional regulator LuxR. LuxR senses AHLs through an AHL-binding region at the amino terminus, enabling LuxR to bind DNA through a helix-turn-helix at the carboxyl terminus to modulate expression of target genes (Nealson et al., 1970; Fuqua et al., 2001; Lupp et al., 2003).

**Figure 2:**
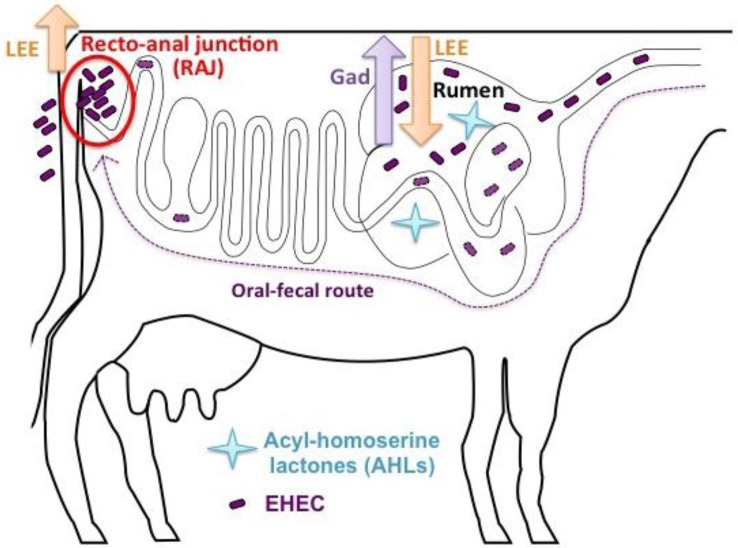
**Proposed EHEC SdiA-AHL signaling in cattle**.

Unlike *V. fischeri*, EHEC lacks a LuxI-like synthase; therefore, SdiA function depends on AHLs produced by other bacteria in the environment (Ahmer et al., 1998). Hughes et al. found that AHLs present in the rumen of cattle activate the *gad* genes that are vital to acid resistance for the passage through the acidic stomachs, and repress the LEE genes to prevent colonization within the rumen. EHEC lacking the SdiA sensor results in significantly reduced acid survival compared to wild type both *in vitro* and in cattle rumen (Hughes et al., 2010). Additionally, wild-type EHEC outcompetes the *sdiA* mutant for colonization at the RAJ (Hughes et al., 2010). Based on these studies, a model has been proposed in which SdiA senses AHLs in rumen to activate acid fitness genes that allow EHEC passage through the acidic stomachs but also downregulate LEE genes to ensure colonization does not occur in hostile environments. EHEC does not encounter AHLs beyond the rumen, alleviating the SdiA-mediated repression of the LEE and allowing EHEC to colonize the RAJ (Figure 2). Intervention in quorum-sensing provides an alternative strategy to reduce carriage in cattle and subsequently, shedding and contamination of EHEC in the environment.

Developing strategies to reduce EHEC survival and colonization in cattle have been an ongoing challenge. Strategies to increase cattle resistance to EHEC colonization include supplementation with probiotics, administration of antibiotics, and vaccination against T3SS machinery (detailed review in Callaway et al., 2009; Jacob et al., 2009). Conflicting results from these studies has thwarted efforts to control EHEC populations within cattle (Potter et al., 2004; Van Donkersgoed et al., 2005; Peterson et al., 2007; Sargeant et al., 2007) and emphasizes the necessity for additional research to be performed. The dearth of knowledge on the mechanisms regulating intestinal colonization of ruminants by EHEC has hindered these strategies. With cattle being the major reservoir of EHEC and bovine-derived products as the prominent source of EHEC outbreaks, understanding the biology of EHEC colonization in cattle is vital to the development of new preventative strategies.

### Conflict of interest statement

The authors declare that the research was conducted in the absence of any commercial or financial relationships that could be construed as a potential conflict of interest.
